# Structural covariance, regional topology, and volumetric aspects of amygdala subnuclei in posttraumatic stress disorder using ultra-high field imaging

**DOI:** 10.1038/s41380-025-03428-9

**Published:** 2025-12-29

**Authors:** Elizabeth M. Haris, Trevor Steward, Kim L. Felmingham, Ben J. Harrison, Christopher G. Davey, Bradford A. Moffat, Rebecca K. Glarin, Richard A. Bryant, Mayuresh S. Korgaonkar

**Affiliations:** 1 Brain Dynamics Centre, Westmead Institute for Medical Research, The University of Sydney, Westmead, NSW Australia; 2 School of Psychology, University of New South Wales, Sydney, Australia; 3 School of Psychological Sciences, Department of Medicine, Dentistry and Health Sciences, The University of Melbourne, Parkville, VIC Australia; 4 Department of Psychiatry, The University of Melbourne, Parkville, Victoria Australia; 5 The Melbourne Brain Centre Imaging Unit, Department of Radiology, The University of Melbourne, Parkville, Victoria Australia; 6 Discipline of Psychiatry, Sydney Medical School, Westmead, NSW Australia; 7 Department of Radiology, Western Sydney Local Health District, Westmead, NSW Australia

**Keywords:** Neuroscience, Psychiatric disorders

## Abstract

The amygdala is a subcortical brain structure involved in threat processing and implicated in various psychopathology. Previous efforts to map amygdala subnuclei connectivity have been hindered by technological limitations. This study used ultra-high field imaging to investigate the covariance profiles of amygdala subnuclei to better understand their contribution to trauma-related psychopathology and posttraumatic stress disorder (PTSD). Participants included 59 non-trauma-exposed controls (NEC; 51% female), 78 trauma-exposed controls (TEC; 65% female), and 73 individuals with PTSD (93% female) who completed T1-weighted MP2RAGE anatomical scans using a 7-Tesla MRI scanner. FreeSurfer was used to parcellate 105 brain regions including nine bilateral amygdala subnuclei. Pearson’s *r* correlations were computed for each subnuclei-brain region pair, corrected for age, sex, education, and total brain volume. Gray matter volumes, topological connectivity (nodal degree) using graph analysis, and subnuclei-brain region covariances were compared between-groups. There were between-group volumetric differences for the lateral nuclei (left: NEC < PTSD/TEC; right: PTSD < NEC/TEC), and higher nodal degree of the right paralaminar subnucleus for TEC (vs NEC). Covariance patterns differed between-groups, with lower PTSD (vs NEC) structural covariances for left cortical and central nuclei, and higher TEC (vs NEC) covariances for left lateral, basal, cortical, and anterior-amygdaloid-area, right cortico-amygdaloid transition, and bilateral paralaminar nuclei. This study is the first to reveal differences in amygdala subnuclei covariance profiles along the trauma-spectrum using ultra-high field imaging. Findings suggest that amygdala subnuclei could have differential connectivity profiles in trauma-related conditions and ultra-high field imaging studies are needed to more precisely understand their role.

## Introduction

The amygdala is a key brain region implicated in posttraumatic stress disorder (PTSD; [1]). Given its role in emotional processing and salience detection [2], understanding the contribution of the amygdala to PTSD is integral to understanding the neurobiology underlying the disorder. The amygdala has long been studied as a homogenous structure in mental health research, particularly in PTSD. Studies examining the structural features of the whole amygdala show mixed findings [3, 4], which might be a consequence of the heterogeneity of the amygdala, a structure which is often delineated into three smaller structures in humans but that can be divided into at least nine substructures using relatively new automated segmentation techniques [5]. One study examining amygdala subnuclei volumes showed smaller bilateral lateral and paralaminar nuclei volumes but larger bilateral central, medial, and cortical nuclei in veterans with PTSD [6]. Another study showed the basal nucleus to be an important hub in the disorder [7], indicating a higher than average number of connections from this subnucleus to other brain regions. A more recent study using a large population sample from the UK Biobank [8] demonstrated the structural coupling of amygdala subregions with areas associated with salience detection (basal ganglia, anterior cingulate) and memory (hippocampus/parahippocampus), highlighting the possibility of differential functional roles of amygdala subnuclei. This is further suggested by functional connectivity studies in PTSD that have shown greater connectivity in PTSD between the basolateral nucleus and dorsal anterior cingulate [9, 10] and middle frontal gyrus [11, 12], brain regions associated with salience detection and the modulation of attention, respectively. Research in rodents and non-human primates demonstrates the contribution of distinct amygdala subnuclei to differential functions ranging from polymodal sensory processing to social, emotional, and reward processing, and different types of memory [13–15].

This evidence highlights the importance of examining the differential profiles of individual amygdala subnuclei to gain a more detailed picture of their underlying connectivity in PTSD. Yet, proper investigation of the role of amygdala subregions in PTSD has been hindered by technical limitations. Specifically, its ventral location and small size (~1.5 cm^3^; [16]) makes it difficult to delineate its substructures using standard field strength 3-Tesla (3 T) magnetic resonance imaging (MRI) scanners currently used in research (due to signal dropout or distortions and a lower signal-to-noise ratio; [17, 18]). The advent of ultra-high field imaging using 7-Tesla (7 T) MRI scanners allows for greater spatial resolution of these structures and higher granularity of amygdala subnuclei and their connectivity profiles [18, 19]. Although magnetic field inhomogeneities are increased at 7 T [18, 19], the finer resolution afforded by anatomical sequences allows for a more detailed examination of the differential contribution of these subnuclei. This could be advantageous to map their detailed neural circuitry in relation to PTSD.

Just as spatially distinct brain regions demonstrate functional connectivity through temporal correlation [20], they also covary in their morphological properties [21]. These inter-individual differences in the structural properties of brain regions at a population level are thought to reflect underlying structural features of remote brain areas that are in some way functionally connected, and is termed structural covariance [21]. Neuropsychiatric research examining structural covariance demonstrates comparable results to functional connectivity research [22], illustrating their complementary nature and representation of underlying brain networks. Examining the structural covariance of amygdala subnuclei in PTSD can further clarify the relationship between these remote structures that might not necessarily be structurally connected. To date, no studies have examined the structural covariance of amygdala subnuclei in PTSD using 7 T.

The aim of this study was to investigate the structural features of amygdala subnuclei using ultra-high field imaging at 7 T. As previous meta-analyses have found that the comparison group mediates neurobiological differences between brain regions [3, 23], we examined the structural covariance profiles of amygdala subnuclei in a sample inclusive of individuals with PTSD in comparison to both healthy individuals with and without prior exposure to trauma. Specifically, we set out to investigate group differences in three domains. First, we examined amygdala subnuclei volumes to delineate any volumetric differences. Second, we assessed regional network topology of amygdala subnuclei relative to the entire brain, and specifically nodal degree to examine the overall connectivity strength of amygdala subnuclei (i.e., nodes that are highly connected with various areas of the brain are deemed to be more important). Third, we measured structural covariance of amygdala subnuclei with individual brain regions to understand the differences in these profiles between groups. Given the improved resolution of ultra-high field imaging, we expected greater sensitivity to detect PTSD and trauma-related differences in amygdala subnuclei volume, topology, and covariance patterns which could have been missed due to imaging at lower field strengths.

## Materials and methods

### Participants

A total of 74 participants with PTSD and 137 controls between the ages of 18–55 were recruited for this study at the University of Melbourne. Of all controls, 78 had experienced traumatic events and were included in the trauma-exposed control (TEC) group, with the remaining categorized as non-trauma-exposed controls (NEC). Exclusions encompassed one PTSD participant and two TEC participants who could not complete their scans, and a NEC participant who experienced technical issues during scanning. This resulted in data available for 59 NEC, 78 TEC, and 73 PTSD participants for analysis (Table 1). Briefly, NEC participants were screened using the Mini-International Neuropsychiatric Interview-7 [24]. Those with PTSD or TEC were pre-screened by being asked if they had been diagnosed with PTSD or exposed to a traumatic experience (war, life-threatening accident, natural disaster, witness to an injury/murder, physical/sexual assault, or torture), followed by a screening using the PTSD Checklist for DSM-5 [25], and the Diagnostic Interview for Anxiety, Mood, and OCD Related Neuropsychiatric Disorders (DIAMOND, version 1.5; [26]). Participants were excluded if they had any neurological/medical conditions for which they were on medication (for example, multiple sclerosis, epilepsy, seizures, etc.), a clinical diagnosis of autism spectrum disorder, bipolar disorder, obsessive-compulsive disorder, schizophrenia, or substance dependence issues, any MRI contraindications, and were not fluent in English. Participants were also excluded if they met criteria for any of these disorders on the DIAMOND. Screening was completed by three researchers trained in administering the DIAMOND. Additional recruitment details can be found in the Supplementary Materials.

**Table 1:** Demographics for non-trauma-exposed controls (NEC), trauma-exposed controls (TEC), and individuals with posttraumatic stress disorder (PTSD).

Demographic Measure	NEC (59)	TEC (78)	PTSD (73)	Stats (*H/W*/*χ*^2^)	*p* value
**Age (Mean (SD))**	21.7 (2.90)	28.0 (8.80)	29.5 (7.6)	44.2	< 0.001 TEC vs PTSD = 0.28 NEC vs TEC/PTSD < 0.001^**^
**Sex (M/F; %)**	29/30 (49/51%)	27/51 (35/65%)	5/68 (7/93%)	30.2	< 0.001 NEC vs TEC = 0.12 PTSD vs NEC/TEC < 0.001^**^
**Education (No/%)**				10.3	< 0.01 NEC vs TEC < 0.01^*^ PTSD vs NEC/TEC < 0.001^**^
Year 10	0 (0.00)	0 (0.00)	1 (1.40)	-	-
Certificate/Diploma	0 (0.00)	12 (15.4)	21 (28.8)	-	-
Trade/Tech School	0 (0.00)	2 (2.56)	14 (19.2)	-	-
Undergrad	34 (57.6)	34 (43.6)	22 (30.1)	-	-
Postgrad	23 (39.0)	28 (35.9)	12 (16.4)	-	-
Other/Prefer not to answer	2 (3.39)	2 (2.56)	3(4.10)	-	-
**Trauma Type (No/%)**				21.5	< 0.001^**^
Natural Disaster	-	17 (21.8)	10 (13.7)	-	-
Accident	-	45 (57.7)	30 (41.1)	-	-
Physical Abuse	-	54 (69.2)	59 (80.8)	-	-
Sexual Abuse	-	23 (29.5)	59 (80.8)	-	-
Trauma experienced before 16 years	-	62 (79.5)	66 (90.0)	-	-
Trauma experienced after 16 years	-	16 (20.5)	7 (9.59)	-	-
**Volumetric Measures**					
Total Brain Volume (M(SD); mm^3^)	1018308 (133437)	1086360 (325001)	1004927 (246426)	5.14	0.08
Left Amygdala Volume	960 (333)	1041 (284)	1093 (261)	5.15	0.08
Right Amygdala Volume	1001 (274)	1099 (263)	1079 (229)	4.33	0.12
Within-Group Amygdala Volume (Left × Right)	-	-	-	1637 (NEC) 2779 (TEC) 2764 (PTSD)	NEC = 0.58 TEC = 0.35 PTSD = 0.70
**PCL-5 Scores (Mean (SD))**	7.80 (7.79)	10.5 (9.74)	49.0 (10.5)	125	< 0.001 NEC vs TEC = 0.22 PTSD vs NEC/TEC < 0.001^**^

*Note*. Statistical tests performed included chi-squared test, and nonparametric Mann-Whitney U (W) and Kruskal-Wallis (H) tests. PCL = PTSD Checklist for DSM-5.

^**^p < 0.001; ^*^p < 0.01.

The study was approved by the University of Melbourne Human Research Ethics Committee (HREC; 2056265) and run in accordance with the Declaration of Helsinki 1975, as revised in 2008. Written informed consent was obtained from all participants, who were also reimbursed on study completion.

### Image acquisition and preprocessing

Neuroimaging was conducted on a 7 T whole body research scanner (Siemens Healthcare, Erlangen, Germany) with 1Tx32R-channel head coil (Nova Medical Inc., Wilmington MA, USA). The protocol for the structural MRI used a self-bias corrected MP2RAGE sequence [27] GR_IR scanning sequence to acquire 3D data comprised of 224 slices, with a sagittal orientation; TR = 5000 ms; TE = 2000ms; flip angle = 4/5 °; in-plane resolution = 0.73mm^3^; matrix = 330 × 330 pixels; slice thickness = 0.73 mm. Full sequence details are available in the Supplement.

**Fig. 1:**
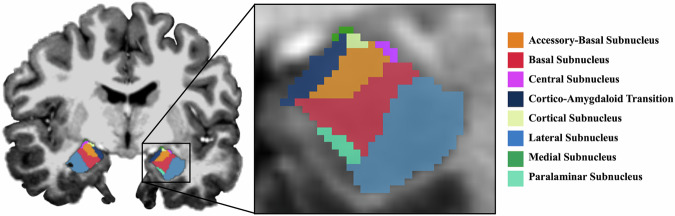
Amygdala subnuclei segmentation on 7 T data using FreeSurfer (version 7.2.0; [28]). Anterior-Amygdaloid-Area not pictured.

Cortical reconstruction and volumetric segmentation of anatomical images was completed using the default processing pipeline from FreeSurfer (version 7.2.0; [28]) on the denoised uniform image (UNIDEN; [29]). This includes motion correction, removal of non-brain tissues, white and gray matter segmentation and boundary definition, intensity normalization, topology correction, and surface deformation. Cortical, subcortical, and amygdala segmentation was performed using the Desikan-Killany-Tourville atlas [30], and automatic subcortical and amygdala segmentation tools in FreeSurfer [5, 28, 31]. Gray matter volumes were extracted for 105 regions: 63 cortical, 24 subcortical, and 18 amygdala subregions (Supplementary Table 1; Fig. 1). We did not directly correct for partial volume effects; however, given that the amygdala segmentation pipeline in FreeSurfer is based on data from a 7 T scanner and that the voxel size of our T1 scans is 0.73mm^3^, this allows for more accurate segmentation, higher signal-to-noise ratio, and finer structural detail that minimizes error associated with partial volume effects. Cortical and subcortical segmentation were inspected to ensure FreeSurfer performed correctly and any outliers for subnuclei volumes—defined as ±3 interquartile range (IQR)—were visually inspected for segmentation failures. A total of four NEC (~7%), eight TEC (~10%), and six PTSD (~8%) participants were flagged as having amygdala subnuclei volumes outside ±3 IQR. Of these, all NEC, three TEC, and two PTSD participants were identified as outliers (i.e., as having multiple volumes (≥4) outside of this range). However, results from analyses conducted with and without the inclusion of these participants remained robust, so they were not removed.

### Statistical analysis

#### Comparison of amygdala subnuclei volumes

Subnuclei volumes between-groups were compared using R [32]. Residuals from a MANOVA investigating overall group differences showed non-normal distribution for most subnuclei volumes. Separate linear mixed effects models were used to 1) examine overall group differences when including all subnuclei in the model:

Volume_ij_ ~ Group_k_ × Subnucleus_j _+ Age + Sex + Education + Total Brain Volume + (1|ID_i_)

and 2) investigate subsequent pairwise group comparisons for each subnuclei to examine these group differences:

NEC × TEC; NEC × PTSD; PTSD × TEC (for each subnucleus; Šidák adjusted; pFWE≤0.05; [33]).

Subject was treated as a random factor, all other variables as fixed factors. Age, sex, total brain volume, and education were included as covariates of no interest. Non-linear age effects did not explain any further variance from the initial linear models used to conduct the analyses. Results for these analyses can be found in the Supplementary Materials.

#### Comparison of amygdala subnuclei structural covariance topology

Graph theory was used to examine the topological properties of amygdala subregions [21]. Specifically, the graph-analysis toolbox (GAT; [34]) was used to compare nodal degree, a measure that designates the importance of a node through defining the total number of connections it has to other nodes.

Pearson’s *r* partial correlation coefficients were derived for each subnuclei-brain region pair (87 in total) to represent covariance networks, and were adjusted for age, sex, total brain volume, and education. Association matrices of 105 × 105 regions per group were constructed, from which undirected binary adjacency matrices were derived, designating values as ‘1’ if exceeding a specific threshold. The lower bound for matrix thresholding was set using the minimum network density at which graphs for all groups were fully connected (D_min_). The upper bound was set at the density at which connections become progressively more random (D_max_), using steps of 2%.

Topological brain networks were evaluated against 20 randomly generated null networks using the Hirschberger-Qi-Steuer algorithm [35]. While the main analysis focused on amygdala subnuclei nodal degree, information on group comparisons on the global topological network measures and hub regions can be found in the Supplementary Materials.

Nonparametric permutation tests using 1000 repetitions and two-sample t-tests were used to compare random networks. Functional data analysis (FDA)—a method the GAT pipeline uses for analyzing data that functions like a curve [34], was used to examine network measures that are calculated at a range of densities. The between-group sum of differences in these network metrics is tested for significance at multiple thresholds (i.e., D_min_ to D_max_) using nonparametric permutations to minimize the effect of numerous group comparisons [36]. To correct for comparisons across multiple nodes, *p-*values were false discovery rate (FDR)-corrected.

#### Comparison of amygdala subnuclei structural covariances between-groups

Pearson’s *r* partial correlations between the gray matter volume of each amygdala subnuclei and every other brain region were used to examine structural covariance. As subnuclei volumes were not normally distributed, correlations were Fisher *z* [37] transformed and compared in separate nonparametric Kruskal-Wallis tests [38] for each subnucleus to examine overall group differences. For step 1, we evaluated the main effect of group (PTSD/TEC/NEC) for all structural covariance measures related to a subnucleus, significant at *p* ≤ 0.02 (0.05/3 group comparisons). If a significant group effect was present for the subnucleus, post-hoc nonparametric pairwise group comparisons (Dunn’s tests; [39, 40]) were conducted to determine which group comparisons to further explore (e.g., PTSD vs TEC, PTSD vs NEC or TEC vs NEC). Lastly, subnucleus-brain region correlations for each significant group comparison from the previous step were compared using the cocor.indep.groups function in R’s cocor package [32, 41, 42], to determine which subnucleus-brain region correlation for that subnucleus was different between groups (i.e., which out of the 87 covariance measures was driving the significant group effect). Fisher *z* statistic and Zou’s confidence intervals are reported [37, 43]. Significant results were FDR-corrected within each group comparison (for 87 regions) using the Benjamini-Hochberg procedure [44].

Analysis code available on request.

## Results

### Sample characteristics

Groups differed on age, sex, and education variables (Table 1). Age and education differences were driven by a significantly younger and more highly educated NEC sample, while sex differences were driven by a primarily female PTSD sample. There was no difference between-groups for total brain volume or whole amygdala volume, nor within-groups when comparing left vs right amygdala volume. Though trauma groups both experienced high levels of childhood trauma they differed in the types of trauma experienced, with the PTSD group showing greater proportion of sexual abuse relative to TEC.

Given that our research question focused on group differences, we included demographic variables as confounds in the following analyses. However, due to the observed demographic imbalances between groups, we also conducted additional exploratory analyses. These included only the females in the sample, as well as structural covariance analyses within the PTSD group using a median split based on age and education. These analyses investigated any potential confounding effects not fully addressed in the main models. A summary of findings is provided below, with the full results included at the end of the Supplementary Materials (Exploratory Analyses).

### Comparison of amygdala subnuclei volumes

A linear mixed effects model examining overall group differences showed an interaction between group and subnuclei (*F*(34,3519) = 1.60; *p* = 0.02). Post-hoc pairwise group comparisons using the estimated marginal means approach (adjusting for the various factors in a linear model; [45]) revealed group differences only for the lateral nucleus (left lateral nucleus: NEC < PTSD and TEC; right lateral nucleus: PTSD < NEC and TEC; Table 2). Other group differences were not significant (*p* > 0.05).

**Table 2:** Significant volumetric differences of subnuclei between-groups.

Amygdala Subregion	Group Contrast	Group Effect	Group Means (volume, mm^3^; Mean (SD))			
			Group 1		Group 2	
Left Lateral Subnucleus	NEC vs TEC	*t*(1546) = −3.45; *p*_*FWE*_ = 0.002 TEC > NEC	NEC:	515 (146)	TEC:	546 (112)
	NEC vs PTSD	*t*(1086) = −3.23; *p*_*FWE*_ = 0.004 PTSD > NEC	NEC:	515 (146)	PTSD:	551 (90)
Right Lateral Subnucleus	NEC vs PTSD	*t*(1086) = 3.36; *p*_*FWE*_ = 0.003 PTSD < NEC	NEC:	563 (84)	PTSD:	539 (71)
	PTSD vs TEC	*t*(1568) = −3.28; *p*_*FWE*_ = 0.003 PTSD < TEC	PTSD:	539 (71)	TEC:	560 (99)

All *p* values significant at *p*_FWE_ ≤ 0.05, using the Šidák correction. Raw group mean volumes are reported.

*NEC* non-trauma-exposed controls, *TEC* trauma-exposed controls, *PTSD* posttraumatic stress disorder.

### Comparison of amygdala subnuclei structural covariance topology

FDA analysis showed TEC (vs NEC) had significantly higher nodal degree for the right paralaminar nucleus (*p*_*FDR*_ = 0.05). No differences in nodal degree were found between PTSD and either control group (uncorrected amygdala subnuclei results can be found in the Supplementary Materials/Supplementary Table 2). Whole-brain network measures showed lower global path length and nodal betweenness, as well as higher global efficiency at minimum density, in PTSD vs NEC/TEC, and TEC vs NEC (Supplementary Table 3; Supplementary Fig. 2).

### Comparison of amygdala subnuclei structural covariances

Initial between-group Kruskal-Wallis tests showed subnuclei connectivity to other brain regions differed between-groups for all but the bilateral medial nuclei and left cortico-amygdaloid-transition (CAT; Table 3). Post-hoc pairwise group comparisons showed results for left hemisphere subnuclei were driven by group differences between NEC and both PTSD and TEC groups, while for right hemisphere subnuclei most effects were driven by differences in all group comparisons (Table 3).

**Table 3:** Group differences for average subnuclei-whole-brain structural covariances and underlying pairwise comparisons driving these overall group differences.

Subnucleus	*p* value (Kruskal Wallis)	Significant Group Comparison	Fisher *Z*	*p* value (Dunn)
Left Lateral	0.004	NEC – PTSD	−2.88	0.002
		NEC – TEC	−2.89	0.002
Left Basal	0.008	NEC – PTSD	−2.09	0.018
		NEC – TEC	−3.03	0.001
Left Accessory-Basal	0.008	NEC – PTSD	−2.28	0.011
		NEC – TEC	−2.99	0.001
Left Anterior-Amygdaloid-Area	<0.001	NEC – PTSD	−3.19	0.001
		NEC – TEC	−3.58	<0.001
Left Central	0.001	NEC – PTSD	−3.09	0.001
		NEC – TEC	−3.23	0.001
Left Medial	0.269 (n.s.)	–	–	–
Left Cortical	0.008	NEC – PTSD	−2.68	0.004
		NEC – TEC	−2.74	0.003
Left Cortico-Amygdaloid Transition	0.145 (n.s.)	–	–	–
Left Paralaminar	0.005	NEC – PTSD	−2.05	0.020
		NEC – TEC	−3.25	0.001
Right Lateral	<0.001	NEC – PTSD	−5.16	<0.001
		PTSD – TEC	−5.05	<0.001
Right Basal	<0.001	NEC – PTSD	−1.71	0.04
		NEC – TEC	−6.38	<0.001
		PTSD – TEC	−4.67	<0.001
Right Accessory-Basal	<0.001	NEC – PTSD	−2.22	0.013
		NEC – TEC	−6.00	<0.001
		PTSD – TEC	−3.78	<0.001
Right Anterior-Amygdaloid-Area	<0.001	NEC – PTSD	1.97	0.025
		NEC – TEC	−3.16	0.001
		PTSD – TEC	−5.13	<0.001
Right Central	0.018	NEC – TEC	−2.84	0.002
Right Medial	0.092 (n.s.)	–	–	–
Right Cortical	<0.001	NEC – PTSD	−3.03	0.001
		NEC – TEC	−4.50	<0.001
Right Cortico-Amygdaloid Transition	<0.001	NEC – PTSD	−3.12	0.001
		NEC – TEC	−6.84	<0.001
		PTSD – TEC	−3.72	<0.001
Right Paralaminar	<0.001	NEC – PTSD	−2.78	0.003
		NEC – TEC	−7.43	<0.001
		PTSD – TEC	−4.64	<0.001

*Note*. Kruskal-Wallis and Dunn tests were used as nonparametric equivalents of ANOVA due to non-normal distribution of the dependent variable. Kruskal-Wallis group effects were significant at *p* ≤ 0.02. Dunn tests were conducted to determine which group comparisons needed further exploration (significant at *p* ≤ 0.05).

*n.s*. not significant, *L* left, *R* right, *NEC* non-trauma-exposed controls, *TEC* trauma-exposed controls, *PTSD* posttraumatic stress disorder.

**Fig. 2:**
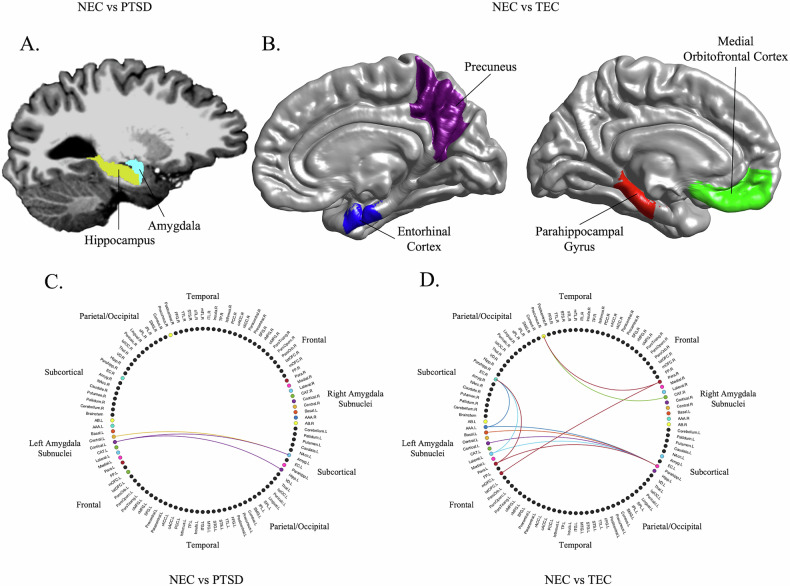
Significant amygdala subnuclei covariance results between groups. **A/C** The non-trauma-exposed controls (NEC) showed higher covariance of amygdala subnuclei with the left hippocampus and amygdala relative to those with posttraumatic stress disorder (PTSD). **B/D** Trauma-exposed controls (TEC) showed higher covariance of amygdala subnuclei with the left parahippocampal gyrus and medial orbitofrontal cortex, and right entorhinal cortex and precuneus relative to NEC. No significant results were observed between PTSD and TEC groups.

Subsequent pairwise group comparisons for each subnucleus were conducted to explore which subnucleus-brain region pairs were underlying these group differences (Table 4; Fig. 2). Non-trauma-exposed controls (vs PTSD) were found to have *higher* structural covariances between the left central nucleus and left amygdala, and between the left cortical nucleus and left amygdala and left hippocampus. In contrast, NEC (vs TEC) were found to have *lower* structural covariances between the left lateral, basal, anterior-amygdaloid-area (AAA), cortical, and paralaminar nuclei and the left parahippocampal gyrus and right entorhinal cortex, and between the right CAT and paralaminar nucleus and the left medial orbitofrontal cortex (mOFC) and right precuneus. Group differences in pairwise structural covariances between TEC and PTSD did not withstand multiple comparison corrections (uncorrected results in Supplementary Table 4).

**Table 4:** Significant structural covariances between subnuclei-brain region pairs driving between-group differences for each individual subnucleus.

Group Comparison	Amygdala Nucleus	Brain Region	*r*1 (NEC)	*r*2 (PTSD/TEC)	Fisher *Z*	FDR-corrected *p* value
NEC > PTSD	Left Central Nucleus	Left Amygdala	0.91	0.70	3.66	0.03
	Left Cortical Nucleus	Left Amygdala	0.91	0.76	2.94	0.04
		Left Hippocampus	0.89	0.72	2.92	0.04
NEC < TEC	Left Lateral Nucleus	Left Parahippocampal Gyrus	−0.60	0.02	−4.07	0.01
		Right Entorhinal Cortex	−0.26	0.34	−3.55	0.02
	Left Basal Nucleus	Left Parahippocampal Gyrus	−0.60	−0.02	−3.85	0.01
	Left Anterior-Amygdaloid-Area	Left Parahippocampal Gyrus	−0.59	0.05	−4.12	<0.01
		Right Entorhinal Cortex	−0.28	0.32	−3.48	0.03
	Left Cortical Nucleus	Left Parahippocampal Gyrus	−0.55	0.01	−3.57	0.04
	Left Paralaminar Nucleus	Left Parahippocampal Gyrus	−0.62	−0.02	−3.96	0.01
		Right Entorhinal Cortex	−0.27	0.31	−3.35	0.04
	Right Cortico-Amygdaloid Transition	Right Precuneus	−0.28	0.35	−3.74	0.02
	Right Paralaminar Nucleus	Left Medial Orbitofrontal Cortex	−0.33	0.26	−3.50	0.03
		Right Precuneus	−0.30	0.32	−3.66	0.03

*Note*. Group differences between TEC and PTSD did not withstand corrections for multiple comparisons.

*r* = Pearson’s partial correlation corrected for age, sex, total brain volume, and education. *r*1 and *r*2 represent the correlation between the amygdala subnucleus and brain region for the NEC group and PTSD or TEC group, respectively. *p* values were false-discovery rate (FDR) corrected for 87 comparisons using the Benjamini-Hochberg method and are significant at *p*_FDR_ ≤ 0.05.

## Exploratory analyses

### Females only

While the effect of sex was controlled for in the above analyses, we also conducted duplicate exploratory analyses including only females, given the substantial imbalance of females across groups. Considering the disproportionate loss of individuals from each group (NEC: 50%; TEC: 35%; PTSD: 7%), and largely unequal group sizes (NEC: 30; TEC: 51; PTSD: 68), these results should be interpreted with caution. A summary of the main findings and a brief discussion are included herein, with complete results included in the Supplementary Materials.

Unlike in the main analysis, FDR-corrected FDA showed significant results for NEC vs TEC for small-worldness: for densities from 0.2–0.5, TEC showed lower small-worldness than NEC. Lower small-worldness indicates that the brain network of TEC is like that of a random network, and as such, does not communicate as efficiently as it would if it were a small-world. Additional global topology results—some similar to the main analysis—are reported in the Supplement. Regional and hub analyses results were not significant when corrected for multiple comparisons; uncorrected results are also reported in the Supplementary Materials.

Structural covariance analyses showed group difference for most subnuclei, with post-hoc group comparisons demonstrating these were largely driven by differences between NEC and both trauma groups (similar to that found above). However, pairwise group comparisons for each subnucleus revealed different subnucleus-brain region pairs responsible for these differences relative to the above analyses. Non-trauma-exposed controls (vs PTSD) were found to have *lower* structural covariances between the left AAA and right insula, left medial nucleus and left posterior cingulate, and the left paralaminar nucleus and left lateral occipital lobe and right insula (*p*_FDR_ ≤ 0.03). As in our main analyses, differences between trauma groups did not survive multiple comparisons. Pairwise comparisons between NEC and TEC also did not survive multiple comparison correction.

Volumetric analyses revealed no differences in amygdala subnuclei volumes between any groups.

### Age

While the effect of age was controlled for in the above analyses, a median split of age in the PTSD group was conducted to explore any structural covariance effects that may be associated with age-related variability. Mean differences between the Young group (median age: 24.5; range 18–29 years) and Old group (median age: 35; range 30–54 years) showed a large effect of *ρ* = 0.67 (Young group mean = 0.73; Old group mean = 0.27; covariances range: −0.68–0.89) for four left hemisphere nuclei and eight different brain regions.

### Education

Similarly, education was controlled for in the main analyses. In the exploratory analysis, a median split within the PTSD group based on highest level of education attained—those with an undergraduate degree or higher versus those without, was conducted to investigate any education-related effects. Differences between the Low education group (N = 39) and High education group (N = 34) were only demonstrated for the right lateral subnucleus and three different brain regions.

Collectively, the effects observed in both age and education exploratory analyses did not overlap with the primary group differences, suggesting that both age and education were not a key contributor to group effects.

## Discussion

This study examined volumetric differences, regional topology, and structural covariance profiles of amygdala subnuclei between NEC, TEC, and individuals with PTSD. Although there were no differences in whole amygdala volumes, PTSD and TEC groups showed significant differences from the NEC group for the left lateral nucleus, while the PTSD group showed significant differences from NEC and TEC groups for the right lateral nucleus. Regional topology analysis identified higher nodal degree for TEC vs NEC for the right paralaminar nucleus. Several subnuclei-brain region pairs showed significant structural covariance results, indicating group differences for areas involved in attention, memory, reward, and salience processing. Collectively, these results reveal important amygdala subnuclei differences in PTSD, and in those who have experienced trauma but have not developed PTSD.

Volumetric differences showed higher left lateral subnuclei volumes for PTSD and TEC groups compared to NEC, but lower right lateral subnuclei volume for PTSD relative to both TEC and NEC groups. It is unclear what this may represent, though prior literature has shown the left amygdala to be associated with fear and threat processing (relative to safety cue processing; [46]), and with sustained emotional processing [47], while the right has been more often associated with automatic and dynamic emotional processing [47, 48]. Meta-analyses have shown volumetric differences between PTSD and TEC groups for the cerebellum [49] and hippocampus [50], but not for the amygdala as a whole [50]. However, smaller bilateral lateral and paralaminar amygdala nuclei have been found in veterans, with larger volumes found for other amygdala subregions [6]. This dissociation between volumes suggests that examining amygdala subnuclei is a more precise way to discern true group differences. The lateral nucleus is the main afferent nucleus of the amygdala, receiving information from polymodal sensory areas [51] and projecting information to other amygdala subnuclei [52] and has been shown to play a role in facilitating the development of PTSD symptomatology [53]. Greater exposure to trauma has been demonstrated as related to greater BLA volume and lower internalizing symptoms during development [54]. Future prospective studies could be used to clarify if higher left lateral subnuclei volumes in PTSD and TEC confer some sort of resilience to traumatic experiences while lower right lateral subnuclei volumes in PTSD mediate some type of worse trauma symptomatology.

In contrast to the volumetric importance of the lateral nucleus, regional network topology showed higher nodal degree for the right paralaminar nucleus in TEC (relative to NEC), indicating that this area has a significantly higher number of connections with other regions of the brain in the TEC group [55]. The few studies investigating PTSD using graph theory often only investigate global network topology and use resting-state fMRI, which make it difficult to discern differences involving amygdala subnuclei [56]. Nevertheless, our results are in line with the one previous study [57] that found the right amygdala to be an important hub in TEC, with our results extending this to show the importance of the right paralaminar nucleus in those with trauma exposure. The paralaminar nucleus consists of a narrow band of densely packed neurons (including immature-appearing neurons that facilitate neurogenesis) that primarily runs alongside the basal nucleus [52, 58]. It has proven elusive to study as it varies in position and prominence across species [14] but is particularly larger in non-human primates and humans than in rodents, suggesting functional importance. It receives projections from the lateral nucleus and hippocampus and is thought to be involved in contextual learning—consolidating past memory information from the hippocampus with present sensory information from the lateral nucleus [14]. Considering that contextual processing facilitates flexible information processing by allowing individuals to derive situationally-informed meanings of the world [59], the importance of the paralaminar nucleus observed in TEC might represent the involvement of contextual processing in resilience to PTSD, and is something to be explored in future research. The lack of any group differences between PTSD and NEC accords with the only previous study that investigated regional topology of amygdala subnuclei in PTSD, albeit in a PTSD and NEC sample scanned on a 3 T scanner [7].

When examining structural covariance, higher left central and cortical nuclei covariance with the left amygdala and higher left cortical nucleus covariance with the left hippocampus was found in NEC relative to PTSD. The central nucleus contains the main efferent connections of the amygdala, is highly connected with the lateral nucleus [52] and receives information about noxious stimuli from the brainstem [60, 61] to facilitate behavioral responding to threatening information. Like the central nucleus, the cortical nucleus also shows numerous connections with the amygdala [62, 63], but also has direct connections to the olfactory system, hippocampus, and parahippocampus that contribute to olfactory-related reward processing in rodents, mice, and humans [13, 52, 64, 65]. In concert, the amygdala receives and facilitates the evaluation and integration of sensory information with long-term, declarative, and episodic memory, which is dependent on the hippocampus [66, 67]. Reduced covariance between the central and cortical nuclei and hippocampus in PTSD might represent dysfunctional odor- and noxious-related stimuli processing in the disorder. Indeed, odor-related processing has been found to be impaired in PTSD, with trauma-related odors enhancing and triggering the recall of emotional memories [68, 69]. Interestingly, in a previous study using 3 T MRI data, we did also find higher covariance between the left central nucleus and whole amygdala in NEC vs PTSD, albeit at an uncorrected level [7]. While this suggests improved sensitivity at 7 T and the potential significance of these subnuclei and types of sensory information to PTSD, it may also suggest a reduction in volume of the left central and cortical nuclei relative to the whole left amygdala in PTSD, particularly considering there were no group differences in overall left amygdala volume in our study and in others [3].

In contrast, structural covariance findings for NEC vs TEC showed lower left lateral, basal, AAA, cortical, and paralaminar nuclei covariance with the left parahippocampal gyrus and right entorhinal cortex for NEC, indicating the importance of connections between regions that are also associated with the hippocampus [13, 70, 71]. A recent study showed parallel volumetric changes in the lateral, cortical, and AAA subnuclei and left parahippocampal gyrus in a large NEC sample [8]. Our results complement and extend these findings, demonstrating greater structural coupling in individuals who have experienced trauma but have not gone on to develop PTSD. Functionally, the multiple subnuclei found to covary with the entorhinal cortex and parahippocampus have also been associated with the integration, moderation, and facilitation of various sensory- and emotionally-driven environmental stimuli to different types of memory [13, 14, 72, 73]. Given that the uncorrected results for PTSD vs TEC also showed higher covariance between the lateral subnucleus and parahippocampus in TEC (Supplementary Table 4), this relationship may represent a neurobiological pathway for trauma resilience. However, this will need further exploration.

In addition, lower structural covariance in NEC (vs TEC) was also found between the right CAT and paralaminar nuclei and left medial OFC and right precuneus. In rodents, the mOFC has been found to innervate lateral and basal nuclei and to regulate value-based responses [74, 75]. More specifically, the mOFC→BLA circuit has been found to mediate environmental cues to understand the value of predicted rewards, while the BLA→mOFC circuit has been found to mediate adaptive responses to cues based on their desirability and predicted reward [76]. The amygdala has also shown connectivity to the precuneus in healthy humans in relation to attentional deployment away from unpleasant aspects of an image [77], indicating the importance of this circuit to emotionally salient information processing. Importantly, the mOFC and precuneus are also part of the default mode network (DMN)—a circuit of the brain that facilitates self-referential processing and non-task based thinking [78], and that has been found to be dysfunctional in TEC [79–81]. It is thought that aberrant integration of the amygdala into the DMN is a key component of this dysfunction [82]. Our results suggest that such dysfunction might be driven by the CAT and paralaminar amygdala subnuclei. Collectively, observed group differences between NEC and PTSD and NEC and TEC (but not between PTSD and TEC) suggest differential covariance profiles for subnuclei that are PTSD or trauma specific, and that warrant further exploration as to their contribution to PTSD proper or to trauma-related psychopathology in general.

Our results need to be considered in terms of the following limitations. Firstly, our data are cross-sectional in nature, preventing any causal inference of results. Secondly, the lack of observed differences between PTSD and TEC samples may be due to the community sample and diverse traumas experienced by our PTSD sample, as opposed to only including individuals who have experienced a single type of trauma, for example veterans, whose results appear more robust [6]. Also, our PTSD group was predominantly female whereas the NEC/TEC group differ in age and/or education profiles relative to the PTSD group. Although age, sex, and education were statistically controlled for in the analyses, these demographic imbalances may still be contributing residual variance that is difficult to fully account for and could potentially be influencing group comparisons. Future work would benefit from exploring the relationships between these confounding factors and the variables of interest, and in comparing homogeneous and diverse trauma samples to determine if these results are in any way mediated by demographic factors or trauma type, timing of trauma (i.e., childhood vs adulthood), or cumulative trauma. Finally, we did not collect detailed information on the comorbidities nor the medications of participants that passed inclusion criteria—information that would be useful to consider in future studies. Importantly, results from the exploratory analyses must also be considered with caution, particularly as this was not part of our original hypothesis. Loss of a significant number of individuals from the NEC group in the females only analyses led to an underpowered sample with groups significantly unequal in sample size, which likely affect the detection of effects, particularly any interaction effects. Stratified group analyses weren’t possible given the unbalanced groups (both in size and variance), and matching participants on unbalanced demographic variables would sacrifice statistical power. Collectively, although results were different for these analyses, these results were exploratory and should not be treated as hypothesis affirming or contradicting, but rather as hypothesis generating in future research.

In summary, this study used ultra-high field neuroimaging to demonstrate the potential importance of the left central, cortical, lateral, basal, AAA, and paralaminar nuclei, and the right CAT and paralaminar nuclei to the neurobiology of PTSD. This might be particularly relevant to the consolidation and reconsolidation of fear memory, odor-related fear processing, visuospatial contextual learning, value-based responding, and self-referential processing associated with the DMN. Furthermore, comparing PTSD, TEC, and NEC allows for a more detailed delineation of these differences along the trauma spectrum, and should be considered in future studies examining amygdala subnuclei and their contribution to PTSD.

## Supplementary information

Supplementry Material

## Data Availability

Data used for this analysis is available upon request.
