# Supplementary material for: Structural covariance, regional topology, and volumetric aspects of amygdala subnuclei in posttraumatic stress disorder using ultra-high field imaging
Source: Mol Psychiatry. 2025 Dec 29;31(5):2954–64. doi: 10.1038/s41380-025-03428-9 (PMC13099380; doi:10.1038/s41380-025-03428-9)
Supplement: Supplementary file 1 — Supplementry Material [file 41380_2025_3428_MOESM1_ESM.docx]

# **Supplementary Information**

# **Materials and Methods**

Recruitment of NEC used online ads, with participants screened using the Mini-International Neuropsychiatric Interview-7 (1) to confirm the absence of mental health disorders. After pre-screening, participants with PTSD/TEC undertook the PTSD Checklist for DSM-5 (PCL-5; 2) and the Diagnostic Interview for Anxiety, Mood, and OCD Related Neuropsychiatric Disorders (DIAMOND, version 1.5; 3), a semi-structured diagnostic interview for DSM-5 disorders. Additionally, trauma-exposure for all participants was assessed using an adapted version of the Life-Stressor Checklist–Revised (4) comprising twelve questions about serious physical and sexual trauma.

A self-bias corrected MP2RAGE sequence (as Siemens WIP925; 5) was implemented for the structural MRI data analyzed in this study. This sequence efficiently obtains 3D T1 weighted volumes at 2 different inversion times. The implementation computes a B1+ inhomogeneity corrected uniform 3D volumetric data set with background noise removal (UNIDEN image; 6). This UNIDEN data set has been previously validated for automated segmentation (6) with MorphoBox (SPM8) to segment 25 brain structures with a mean intra-subject difference of ~6.6%. Although the denoising process introduces a small residual bias field, this is small enough to be further corrected by the bias field correction algorithm implemented in FreeSurfer (7).

**Supplementary Table 1.** Gray matter volumes extracted with FreeSurfer and used as regions of interest (ROI) for all analyses.

| **Amygdala Areas** | **Cortical Areas** | **Subcortical Areas** | |
| --- | --- | --- | --- |
| Accessory Basal Nucleus | L/R Frontal Pole | L/R Thalamus | |
| Anterior-Amygdaloid-Area | L/R Medial Orbitofrontal Cortex | L/R Ventral Diencephalon^1^ | |
| Basal Nucleus | L/R Lateral Orbitofrontal Cortex | L/R Hippocampus | |
| Central Nucleus | L/R Pars Orbitalis | L/R Parahippocampal Gyrus | |
| Cortical Nucleus | L/R Pars Opercularis | L/R Entorhinal Cortex | |
| Cortico-Amygdaloid Transition | L/R Pars Triangularis | L/R Amygdala | |
| Lateral Nucleus | L/R Middle Frontal Area (caudal) | L/R Accumbens | |
| Medial Nucleus | L/R Middle Frontal Area (rostral) | L/R Caudate | |
| Paralaminar Nucleus | L/R Superior Frontal Lobe | L/R Putamen | |
|  | L/R Precentral Gyrus | L/R Pallidum | |
|  | L/R Paracentral Gyrus | L/R Cerebellum | |
|  | L/R Anterior Cingulate Cortex (rostral) | Brain Stem | |
|  | L/R Anterior Cingulate Cortex (caudal) |  | |
|  | L/R Posterior Cingulate Cortex  L/R Isthmus Cingulate |  | |
|  | L/R Temporal Pole |  | |
|  | L/R Insula |  |  |
|  | L/R Inferior Temporal Lobe |  |  |
|  | L/R Middle Temporal Lobe |  |  |
|  | L/R Superior Temporal Lobe |  |  |
|  | L/R Bank Superior Temporal Sulcus |  |  |
|  | L/R Transverse Temporal Lobe |  |  |
|  | L/R Fusiform Gyrus |  |  |
|  | L/R Postcentral Gyrus |  |  |
|  | L/R Precuneus |  |  |
|  | L/R Cuneus |  |  |
|  | L/R Supramarginal Gyrus |  |  |
|  | L/R Inferior Parietal Cortex |  |  |
|  | L/R Superior Parietal Lobe |  |  |
|  | L/R Lingual Gyrus |  |  |
|  | L/R Pericalcarine Fissure |  |  |
|  | L/R Lateral Occipital Lobe |  | |

*Note.* L=left. R=right. ^1^Ventral diencephalon includes the following areas: hypothalamus, mammillary body, subthalamic nuclei, substantia nigra, red nucleus, lateral geniculate nucleus, and medial geniculate nucleus.

# **Results**

## **Comparison of amygdala subnuclei volumes**

A MANOVA including all amygdala subnuclei as dependent variables showed an overall age effect (*p*=.005). However, univariate ANOVAs did not show any one nucleus to be driving this effect, suggesting a distributed or subtle effect of age on subnuclei volume. Non-linear age effects were also explored, however, neither quadratic (MANOVA model age effect: *p*=.01; linear model comparison: *p*=.30) nor cubic (MANOVA model age effect: *p*=.03; linear model comparison *p*=.54) models significantly improved the fit of these multivariate models beyond the linear effect of age (Figure S1).

Linear model: (Volume ~ Group + Age + Sex + Education + Total Brain Volume)

Quadratic model: (Volume ~ Group + Age + Age^2^ + Sex + Education + Total Brain Volume)

Cubic model: (Volume ~ Group + Age + Age^3^ + Sex + Education + Total Brain Volume)

## **Comparison of amygdala subnuclei structural covariance topology**

Graph theory was employed to investigate the topology of amygdala subnuclei as it is a methodology that employs a data-driven approach to characterize covariance patterns amongst brain regions into properties that are biologically meaningful (8). Uncorrected regional topological differences between PTSD and TEC groups were found for nodal betweenness and nodal degree using FDA (Supplementary Table 2). Uncorrected differences between PTSD and NEC were also found for normalized nodal betweenness, showing the right paralaminar nucleus as important in PTSD (*p*=.05). Uncorrected differences between TEC and NEC showed differences in FDR-corrected FDA analyses for nodal betweenness and degree, with higher nodal degree for the right paralaminar nucleus and higher nodal betweenness for the left CAT in TEC (*p*’s=.05).

Hub analysis revealed several important regions for efficient communication at an uncorrected level when comparing both TEC and PTSD and NEC and TEC groups, however, only the left CAT emerged as a significant region for TEC relative to those with PTSD.

Global network measures for group comparisons included clustering coefficient (the interconnection of nodes), characteristic path length (the shortest path between two areas), small-worldness (the balance between clustering and path length), global efficiency (of information transfer across the brain), and local efficiency (of information transfer within smaller brain regions), with FDR-corrected *p*-values Bonferroni corrected to account for all five measures (.05/5=*p*≤.01). Binary adjacency matrices for all three group comparisons can be found in Figure S2, demonstrating differential covariance profiles of amygdala subnuclei between groups. When examining structural covariances at minimum density, differences were found between-groups for characteristic path length (TEC vs PTSD *p*_FWE_=.005; NEC vs PTSD/TEC *p*_FWE_=.001), mean node betweenness (TEC vs PTSD *p*_FWE_=.005; NEC vs PTSD/TEC *p*_FWE_=.001), and global efficiency (all group differences *p*_FWE_<.001). These differences were driven by lower characteristic path length and mean node betweenness in PTSD vs TEC/NEC and in TEC vs NEC; and higher global efficiency in PTSD vs both groups and in TEC vs NEC. Average topological values can be found in Supplementary Table 3.

**Supplementary Figure 1.** Linear, quadratic, and cubic age effects on amygdala subnuclei volume in a multivariate analysis of variance. Effects did not differ from one another (*p*’s>.03).

**Supplementary Table 2.** Uncorrected (*p*<.05) FDA results for regional measures for trauma-exposed controls (TEC) vs posttraumatic stress disorder (PTSD).

| **Regional Measure** | **Group Comparison** | **Region** | ***p* value** |
| --- | --- | --- | --- |
| FDA Nodal Degree | PTSD < TEC | Right Anterior-Amygdaloid-Area | 0.019 |
|  | PTSD < TEC | Right Cortico-Amygdaloid-Transition | 0.023 |
|  | PTSD < TEC | Right Basal Nucleus | 0.036 |
| FDA Nodal Betweenness | PTSD < TEC | Left Cortico-Amygdaloid-Transition | 0.009 |
|  | PTSD < TEC | Left Paralaminar Nucleus | 0.014 |

*Note.* Results were significant when using Functional Data Analysis (FDA), however, they were not significant at an FDR-corrected and family-wise error corrected level for multiple regional measures (*p*≤.02). Nodal Betweenness represents the number of paths crossing a node (indicating importance to efficient information transfer). Nodal Degree represents the number of connections a node has (indicating nodal importance).

**Supplementary Table 3.** Significant group differences in global network topology at minimum density, measured using grey matter volume.

| **Group Comparison** | **Global Network Measure** | **Group 1** | **Group 2** |
| --- | --- | --- | --- |
| PTSD < TEC^*^ | Path length | 1.61 | 1.70 |
| PTSD < NEC^**^ |  | 1.69 | 2.00 |
| PTSD < NEC^**^ | Nodal betweenness | 64.7 | 74.5 |
| PTSD < TEC^*^ |  | 73.2 | 106.2 |
| PTSD > TEC^**^ | Global efficiency | 0.70 | 0.66 |
| PTSD > NEC^**^ |  | 0.67 | 0.58 |
| TEC < NEC^**^ | Path length | 1.77 | 2.16 |
| TEC < NEC^**^ | Nodal betweenness | 82.0 | 122.4 |
| TEC > NEC^**^ | Global efficiency | 0.64 | 0.55 |

Note. NEC=non-trauma-exposed controls. TEC=trauma-exposed controls. PTSD=posttraumatic disorder. Group measures represent average network measure. *p* values were false-discovery rate corrected and adjusted for the five comparisons being measured (.05/5=.01). ^*^Significant at *p*_FWE_≤.005. ^**^Significant at *p*_FWE_≤.001.


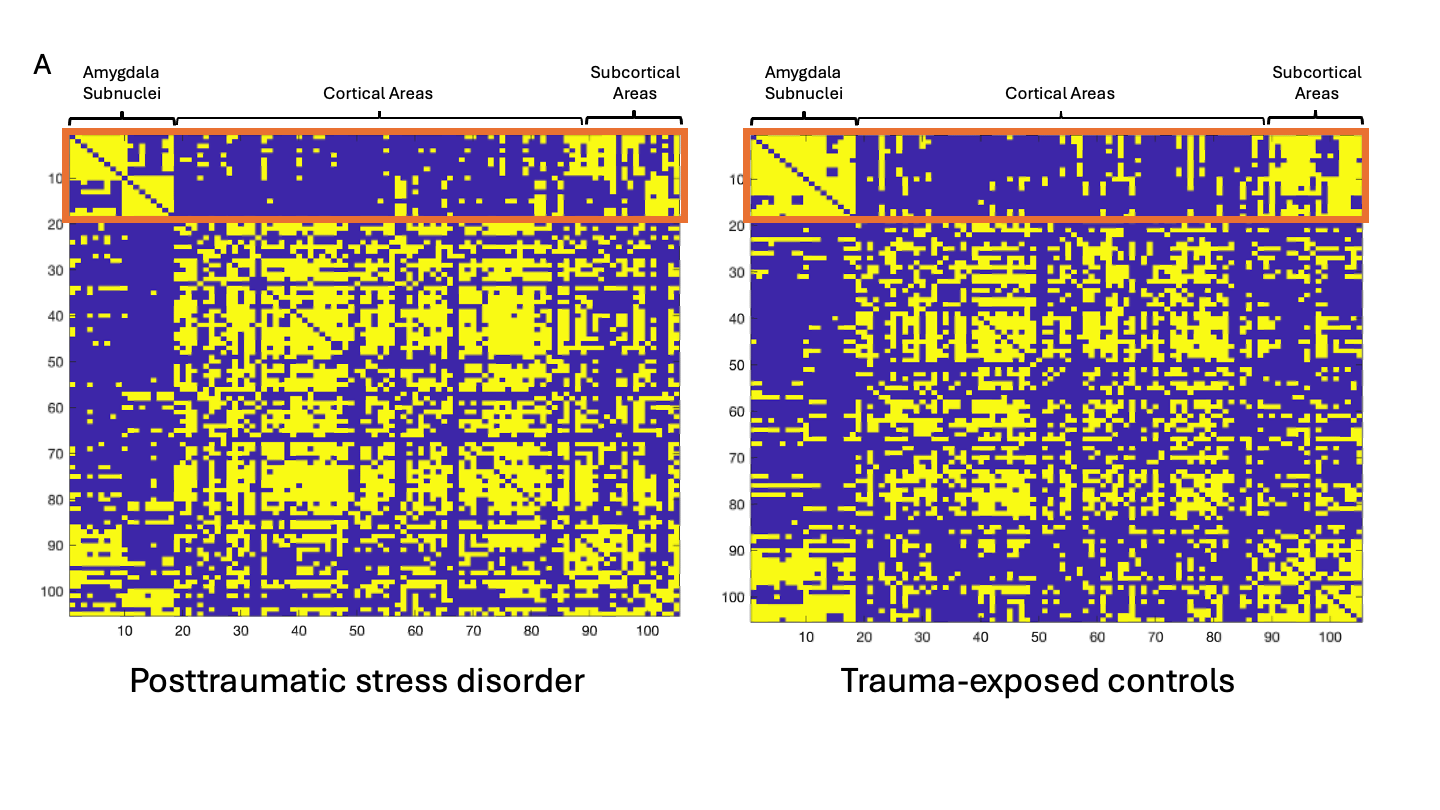


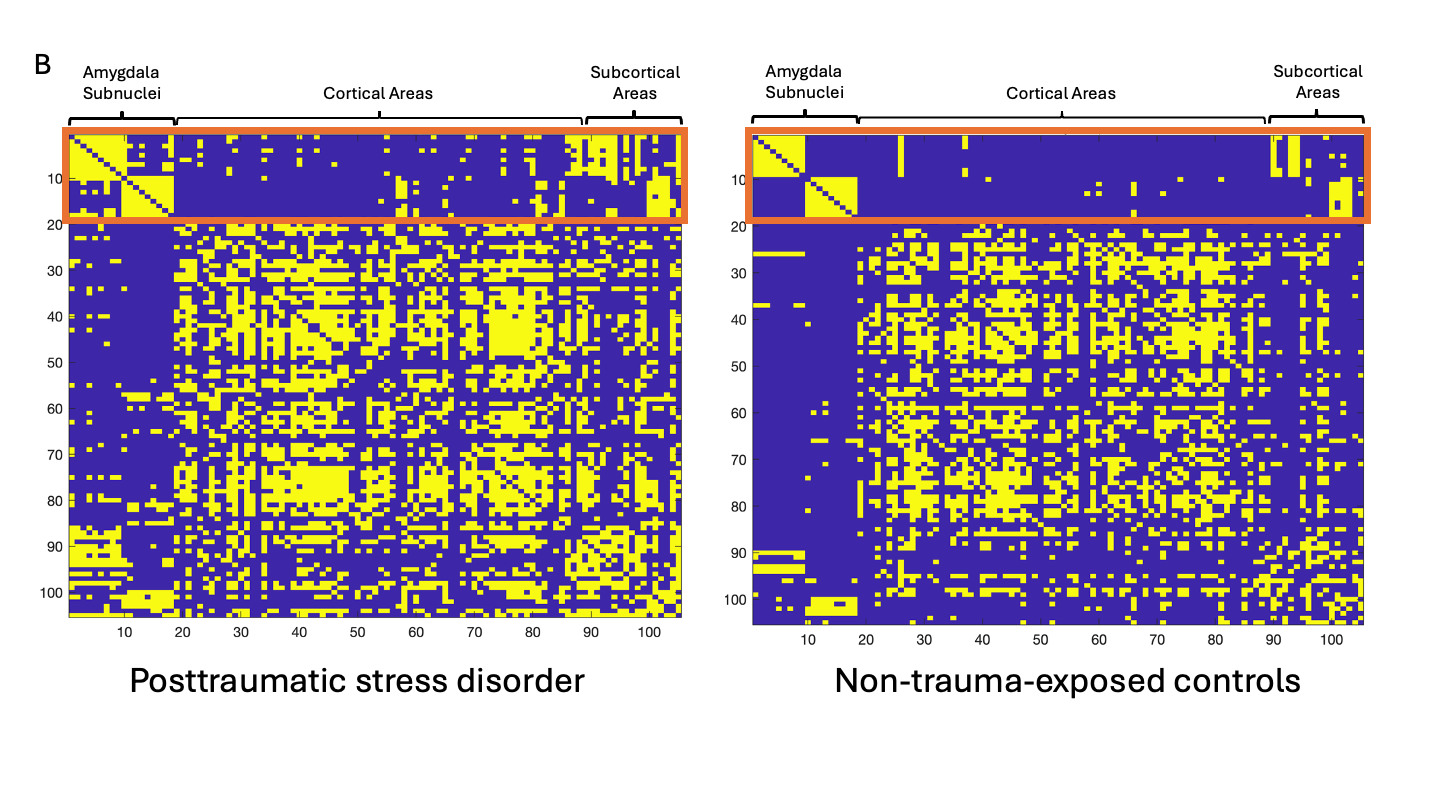


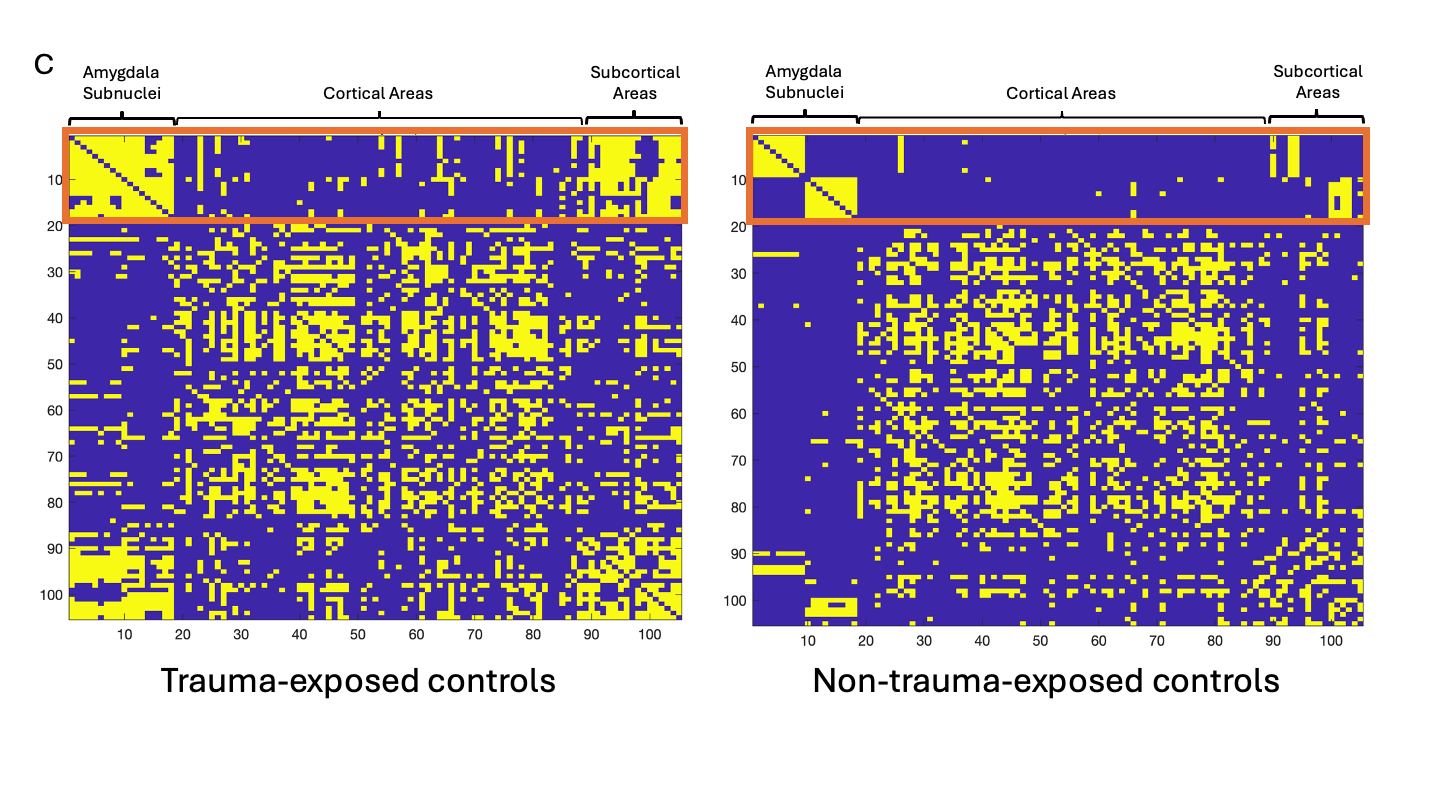


**Supplementary Figure 2.** Binary adjacency matrices for posttraumatic stress disorder (PTSD) vs trauma-exposed controls (TEC; A), PTSD vs non-trauma-exposed controls (NEC; B) and TEC vs NEC (C). The orange box highlights intra-network amygdala subnuclei covariances, and subnuclei covariances with cortical and subcortical areas. Covariances are clearly different between groups, with a pattern of greater number of inter-regional correlations from NEC to TEC to PTSD. Maps were thresholded at D_min_=.41 (TEC vs PTSD), D_min_=.35 (NEC vs PTSD), and D_min_=.30 (NEC vs TEC). Minimum density (D_min_) designates the point at which all nodes within both networks are fully connected.

## **Comparison of amygdala subnuclei structural covariances between-groups (next page)**

**Supplementary Table 4.** Uncorrected pairwise group differences in structural covariances between subnuclei-brain region pairs between trauma-exposed controls (TEC) and individuals with posttraumatic stress disorder (PTSD).

| **Amygdala Nucleus** | **Brain Region** | ***r*1** **(TEC)** | ***r*2 (PTSD)** | **Fisher *Z*** | **Uncorrected*****p* value** |
| --- | --- | --- | --- | --- | --- |
| Right Lateral | Left Lateral Orbitofrontal Cortex | 0.38 | -0.10 | 3.03 | .002 |
|  | Left Superior Temporal Gyrus | 0.40 | 0.01 | 2.48 | .013 |
|  | Left Transverse Temporal Gyrus | 0.30 | -0.07 | 2.31 | .021 |
|  | Right Lingual Gyrus | 0.25 | -0.14 | 2.38 | .017 |
|  | Right Parahippocampal Gyrus | 0.22 | -0.19 | 2.51 | .012 |
|  | Right Lateral Orbitofrontal Cortex | 0.26 | -0.09 | 2.14 | .032 |
|  | Left Superior Frontal Gyrus | 0.35 | 0.03 | 1.98 | .048 |
| Right Basal | Left Anterior-Amygdaloid-Area | 0.44 | 0.13 | 2.03 | .043 |
|  | Left Paralaminar Nucleus | 0.41 | 0.10 | 2.04 | .041 |
|  | Left Precuneus | 0.33 | 0.00 | 2.11 | .035 |
|  | Left Superior Temporal Gyrus | 0.35 | 0.02 | 2.09 | .037 |
|  | Right Inferior Parietal Lobe | 0.08 | -0.28 | 2.19 | .029 |
|  | Right Isthmus Cingulate | 0.29 | -0.12 | 2.47 | .014 |
|  | Left Putamen | 0.38 | -0.01 | 2.48 | .013 |
|  | Left Nucleus Accumbens | 0.23 | -0.12 | 2.16 | .031 |
|  | Left Ventral Diencephalon^1^ | 0.43 | 0.09 | 2.25 | .025 |
| Right Accessory-Basal | Left Anterior-Amygdaloid-Area | 0.42 | 0.05 | 2.39 | .017 |
|  | Left Central Nucleus | 0.48 | 0.13 | 2.34 | .019 |
|  | Right Isthmus Cingulate | 0.24 | -0.16 | 2.40 | .016 |
|  | Left Putamen | 0.42 | 0.01 | 2.62 | .009 |
|  | Left Nucleus Accumbens | 0.22 | -0.17 | 2.40 | .017 |
|  | Left Paralaminar Nucleus | 0.35 | 0.02 | 2.05 | .040 |
|  | Left Precuneus | 0.32 | 0.00 | 1.98 | .048 |
|  | Right Inferior Parietal Lobe | 0.03 | -0.28 | 1.96 | .050 |
|  | Left Hippocampus | 0.42 | 0.11 | 2.01 | .044 |
|  | Left Ventral Diencephalon | 0.36 | 0.02 | 2.13 | .033 |
|  | Right Thalamus | 0.30 | -0.02 | 2.01 | .044 |
| Right Anterior-Amygdaloid-Area | Brain Stem | 0.44 | -0.01 | 2.92 | .003 |
|  | Left Ventral Diencephalon | 0.45 | 0.00 | 2.88 | .004 |
|  | Left Lateral Nucleus | 0.51 | 0.16 | 2.45 | .014 |
|  | Left Paralaminar Nucleus | 0.46 | 0.07 | 2.52 | .012 |
|  | Left Hippocampus | 0.46 | 0.10 | 2.44 | .015 |
|  | Left Nucleus Accumbens | 0.24 | -0.16 | 2.48 | .013 |
|  | Left Anterior-Amygdaloid-Area | 0.52 | 0.21 | 2.15 | .031 |
|  | Left Middle Temporal Gyrus | 0.20 | -0.16 | 2.16 | .030 |
|  | Right Thalamus | 0.32 | -0.02 | 2.14 | .033 |
|  | Left Basal Nucleus | 0.46 | 0.15 | 2.05 | .040 |
|  | Right Posterior Cingulate | 0.15 | -0.19 | 2.03 | .042 |
| Right Cortico-Amygdaloid Transition | Left Anterior-Amygdaloid-Area | 0.46 | 0.17 | 1.97 | .049 |
|  | Right Isthmus Cingulate | 0.27 | -0.07 | 2.13 | .033 |
|  | Right Precuneus | 0.35 | -0.01 | 2.27 | .023 |
|  | Left Putamen | 0.41 | 0.05 | 2.38 | .018 |
|  | Brain Stem | 0.37 | 0.03 | 2.18 | .029 |
|  | Left Ventral Diencephalon | 0.49 | 0.12 | 2.49 | .013 |
| Right Paralaminar | Left Inferior Parietal Lobe | 0.28 | -0.05 | 2.06 | .040 |
|  | Left Lateral Orbitofrontal Cortex | 0.22 | -0.11 | 1.99 | .046 |
|  | Left Pars Triangularis | 0.23 | -0.12 | 2.12 | .034 |
|  | Left Precuneus | 0.37 | -0.04 | 2.56 | .011 |
|  | Left Superior Temporal Gyrus | 0.37 | 0.03 | 2.18 | .029 |
|  | Right Inferior Parietal Lobe | 0.12 | -0.21 | 1.99 | .046 |
|  | Right Isthmus Cingulate | 0.28 | -0.04 | 2.01 | .045 |
|  | Right Lingual Gyrus | 0.18 | -0.15 | 1.97 | .049 |
|  | Right Precuneus | 0.32 | -0.09 | 2.55 | .011 |
|  | Brain Stem | 0.41 | 0.08 | 2.10 | .035 |
|  | Left Ventral Diencephalon | 0.45 | 0.14 | 2.08 | .038 |

*Note.* ^1^Ventral diencephalon includes the following areas: hypothalamus, mammillary body, subthalamic nuclei, substantia nigra, red nucleus, lateral geniculate nucleus, and medial geniculate nucleus. *r*=Pearson’s partial correlation corrected for age, sex, and total brain volume. *r*1 and *r*2 represent the correlation between the amygdala nucleus and brain region for the TEC and PTSD group, respectively.

# **Exploratory Analysis**

## **Females Only**

## **Comparison of amygdala subnuclei structural covariance topology**

At minimum density, higher global and local efficiency was demonstrated in PTSD (vs TEC/NEC), higher clustering was shown in PTSD (vs TEC) and in TEC (vs NEC), and lower characteristic path length and nodal betweenness were demonstrated in PTSD (vs TEC) and in PTSD/TEC (vs NEC). All results were *p*<.01, and Bonferroni corrected to account for the five measures of interest (.05/5=*p*≤.01). Of note, higher global efficiency in PTSD and lower characteristic path length in PTSD and TEC were similar to results found in the main analysis.

Also similar to the main analysis, FDR-corrected FDA results for regional topological measures were not significant for any group. Uncorrected (*p*≤.05) results showed higher nodal degree for the right lateral subnucleus in PTSD (vs TEC; *p*=.05), higher nodal degree for the right CAT and paralaminar subnuclei and higher nodal betweenness for the right CAT in PTSD (vs NEC; *p*’s=.01), and higher clustering, nodal betweenness and nodal degree for several nodes in TEC (vs NEC): clustering (right central (*p*=.03) and cortical subnuclei (*p*=.01)), nodal betweenness (left AAA (*p*=.03)), and nodal degree (left lateral, basal, AB, AAA, and PL subnuclei (*p*’s≤.04), and right CAT (*p*=.01). Lower nodal degree was also found for the right lateral subnucleus in TEC (vs NEC; *p*=.01). Notably, only one of these results (underlined) was also found in the main analysis.

Several areas were shown to be important hubs in all analyses. However, FDA only showed the right lateral subnucleus as important for NEC and TEC (vs PTSD) for nodal betweenness (which was different to the results obtained in the main analysis).

## **Comparison of amygdala subnuclei structural covariances**

Initial between-group Kruskal-Wallis tests showed subnuclei connectivity to other brain regions differed between-groups for all except the right anterior-amygdaloid-area, central, medial, and cortical nuclei. Post-hoc pairwise group comparisons showed results were largely driven by group differences between NEC and both trauma groups (Supplementary Table 5).

Subsequent pairwise group comparisons for each subnucleus were conducted to explore which subnucleus-brain region pairs were underlying these group differences (Supplementary Table 6). Non-trauma-exposed controls (vs PTSD) were found to have *lower* structural covariances between the left anterior-amygdaloid-area and right insula, left medial nucleus and left posterior cingulate, and the left paralaminar nucleus and left lateral occipital lobe and right insula. Group differences in pairwise structural covariances between TEC and NEC/PTSD groups did not withstand multiple comparison corrections.

**Supplementary Table 5.** Group differences for average subnuclei-whole-brain structural covariances and underlying pairwise comparisons driving overall group differences for females only.

| **Subnucleus** | ***p* value**  **(Kruskal Wallis)** | **Significant**  **Group Comparison** | **Fisher *Z*** | ***p* value**  **(Dunn)** |
| --- | --- | --- | --- | --- |
| Left Lateral | <.001 | NEC – PTSD | -5.29 | <.001 |
|  |  | NEC – TEC | -5.28 | <.001 |
| Left Basal | <.001 | NEC – PTSD | -5.27 | <.001 |
|  |  | NEC – TEC | -5.77 | <.001 |
| Left Accessory-Basal | <.001 | NEC – PTSD | -5.74 | <.001 |
|  |  | NEC – TEC | -5.97 | <.001 |
| Left Anterior-Amygdaloid-Area | <.001 | NEC – PTSD | -5.61 | <.001 |
|  |  | NEC – TEC | -6.18 | <.001 |
| Left Central | .001 | NEC – PTSD | -6.21 | <.001 |
|  |  | NEC – TEC | -5.37 | <.001 |
| Left Medial | <.001 | NEC – PTSD | -4.82 | <.001 |
|  |  | NEC – TEC | -3.46 | <.001 |
| Left Cortical | <.001 | NEC – PTSD | -5.80 | <.001 |
|  |  | NEC – TEC | -5.13 | <.001 |
| Left Cortico-Amygdaloid Transition | <.001 | NEC – PTSD | -4.55 | <.001 |
|  |  | NEC – TEC | -4.66 | <.001 |
| Left Paralaminar | <.001 | NEC – PTSD | -5.24 | <.001 |
|  |  | NEC – TEC | -6.27 | <.001 |
| Right Lateral | <.001 | NEC – TEC | -5.45 | <.001 |
|  |  | PTSD – TEC | -4.34 | <.001 |
| Right Basal | <.001 | NEC – PTSD | -3.40 | <.001 |
|  |  | NEC – TEC | -6.04 | <.001 |
|  |  | PTSD – TEC | -2.64 | <.001 |
| Right Accessory-Basal | <.001 | NEC – PTSD | -2.30 | .011 |
|  |  | NEC – TEC | -4.65 | <.001 |
|  |  | PTSD – TEC | -2.35 | .010 |
| Right Anterior Amygdaloid Area | .05 (n.s.) | – | – | – |
| Right Central | .03 (n.s.) | – | – | – |
| Right Medial | .19 (n.s.) | – | – | – |
| Right Cortical | .30 (n.s.) | – | – | – |
| Right Cortico-Amygdaloid Transition | <.001 | NEC – PTSD | -4.98 | <.001 |
|  |  | NEC – TEC | -6.40 | <.001 |
| Right Paralaminar | <.001 | NEC – PTSD | -5.64 | <.001 |
|  |  | NEC – TEC | -7.60 | <.001 |

*Note.* Kruskal-Wallis and Dunn tests were used as nonparametric equivalents of ANOVA due to non-normal distribution of the dependent variable. Kruskal-Wallis group effects were significant at *p*≤.02. Dunn tests were conducted to determine which group comparisons needed further exploration (significant at *p*≤.05). n.s.=not significant. L=left. R=right. NEC=non-trauma-exposed controls. TEC=trauma-exposed controls. PTSD=posttraumatic stress disorder.

**Supplementary Table 6.** Significant structural covariances between subnuclei-brain region pairs driving between-group differences for each individual subnucleus for females only. Group differences between TEC and NEC/PTSD did not withstand corrections for multiple comparisons.

| **Group Comparison** | **Amygdala Nucleus** | **Brain Region** | ***r*1 (NEC)** | ***r*2 (PTSD)** | **Fisher *Z*** | **FDR-corrected**  ***p* value** |
| --- | --- | --- | --- | --- | --- | --- |
| NEC < PTSD | Left Anterior-Amygdaloid Area | Right Insula | -0.37 | 0.42 | -3.68 | .03 |
|  | Left Medial Nucleus | Left Posterior Cingulate | -0.42 | 0.42 | -3.92 | .009 |
|  | Left Paralaminar Nucleus | Left Lateral Occipital Lobe | -0.43 | 0.33 | -3.49 | .03 |
|  |  | Right Insula | -0.40 | 0.35 | -3.42 | .03 |

*Note.* *r*=Pearson’s partial correlation corrected for age, total brain volume, and education. *r*1 and *r*2 represent the correlation between the amygdala subnucleus and brain region for the NEC and PTSD groups, respectively. *p* values were false-discovery rate (FDR) corrected for 87 comparisons using the Benjamini-Hochberg method and are significant at *p*_FDR_≤.05.

## **Age**

Group differences between Young and Old (median split) PTSD participants showed mean covariances that were *higher* in the Young group and *lower* in the Old group for four subnuclei in the left hemisphere. Differences were found for frontal, temporal, and subcortical areas largely involved in motor and memory processes. All results can be found in Supplementary Table 7 and Supplementary Figure 3.

## **Education**

Group differences between Low and High education (high = undergraduate+) PTSD participants showed mean covariances that were higher in the High education group for the right lateral subnucleus and brain areas in the frontal lobe associated with somatosensory process, and higher in the Low education group for the parahippocampal gyrus, an area associated with memory. All results can be found in Supplementary Table 8.

**Supplementary Table 7.** Covariance results representing group differences between Young and Old (median split) PTSD participants.

| **Amygdala Subnucleus**  **(Left Hemisphere)** | **Brain Region** | **Fisher Z** | **FDR-corrected *p* value** | **r1 (Young)** | **r2**  **(Old)** |
| --- | --- | --- | --- | --- | --- |
| Lateral Nucleus | Left Hippocampus | 3.06 | 0.04 | 0.79 | 0.31 |
| Basal Nucleus | Right Precentral Gyrus | 3.44 | 0.02 | 0.39 | -0.42 |
| Basal Nucleus | Left Hippocampus | 3.13 | 0.04 | 0.89 | 0.57 |
| Basal Nucleus | Left Pallidum | 3.07 | 0.04 | 0.47 | -0.24 |
| Cortico-amygdaloid Transition | Left Hippocampus | 3.48 | 0.01 | 0.86 | 0.42 |
| Cortico-amygdaloid Transition | Right Insula | 3.18 | 0.03 | 0.38 | -0.36 |
| Medial Nucleus | Left Inferior Temporal Gyrus | 3.98 | 0.01 | 0.16 | -0.68 |
| Medial Nucleus | Left Entorhinal Cortex | 3.43 | 0.02 | 0.34 | -0.46 |
| Medial Nucleus | Right Cerebellum | 3.50 | 0.02 | 0.07 | -0.66 |
| Medial Nucleus | Left Cerebellum | 3.23 | 0.03 | 0.19 | -0.54 |

*Note.* *r*=Pearson’s partial correlation corrected for sex, total brain volume, and education. *r*1 and *r*2 represent the correlation between the amygdala subnucleus and brain region for the Young and Old PTSD groups, respectively. *p* values were false-discovery rate (FDR) corrected for 87 comparisons using the Benjamini-Hochberg method and are significant at *p*_FDR_≤.05.

**Supplementary Figure 3.** The effects of age on structural covariances of amygdala subnuclei. Median split of posttraumatic stress group into young (M=24.5) and old ages (M=35). Mean group difference in covariance denotes a large effect at *r*=0.67 with covariances ranging from 0.27 (Old group) to 0.73 (Young group), and corrected for sex, education, and total brain volume.

**Supplementary Table 8.** Covariance results representing group differences between Low and High Education (High = Undergraduate+) PTSD participants.

| **Amygdala Subnucleus**  **(Right Hemisphere)** | **Brain Region** | **Fisher Z** | **FDR-corrected *p* value** | **r1 (Low)** | **r2**  **(High)** |
| --- | --- | --- | --- | --- | --- |
| Lateral Nucleus | Right Postcentral Gyrus | -3.74 | 0.02 | -0.33 | 0.52 |
| Lateral Nucleus | Left Postcentral Gyrus | -3.31 | 0.03 | -0.41 | 0.37 |
| Lateral Nucleus | Right Parahippocampal Gyrus | 3.32 | 0.03 | 0.16 | -0.57 |

*Note.* *r*=Pearson’s partial correlation corrected for sex, total brain volume, and age. *r*1 and *r*2 represent the correlation between the amygdala subnucleus and brain region for the Low Education and High Education PTSD groups, respectively. *p* values were false-discovery rate (FDR) corrected for 87 comparisons using the Benjamini-Hochberg method and are significant at *p*_FDR_≤.05.

## **References**

1. Sheehan DV, Lecrubier Y, Sheehan KH, Amorim P, Janavs J, Weiller E, et al. The Mini-International Neuropsychiatric Interview (M.I.N.I.): the development and validation of a structured diagnostic psychiatric interview for DSM-IV and ICD-10. *J Clin Psychiatry*. 1998;59 Suppl 20:22-33;quiz 4-57.

2. Weathers FW, Litz BT, Keane TM, Palmieri PA, Marx BP, Schnurr PP. The PTSD Checklist for DSM-5 (PCL-5). Scale available from the National Center for PTSD at <www.ptsd.va.gov>. 2013.

3. Tolin DF, Gilliam C, Wootton BM, Bowe W, Bragdon LB, Davis E, et al. Psychometric Properties of a Structured Diagnostic Interview for DSM-5 Anxiety, Mood, and Obsessive-Compulsive and Related Disorders. *Assessment*. 2018;25(1):3-13.

4. Wolfe J, Kimerling R, Brown PJ, Chrestman KR, Levin K. Life Stressor Checklist-Revised (LSC-R). *APA PsycTests*. 1996.

5. Marques JP, Kober T, Krueger G, van der Zwaag W, Van de Moortele P-F, Gruetter R. MP2RAGE, a self bias-field corrected sequence for improved segmentation and T1-mapping at high field. *NeuroImage*. 2010;49(2):1271-81.

6. O'Brien KR, Kober T, Hagmann P, Maeder P, Marques J, Lazeyras F, et al. Robust T1-Weighted Structural Brain Imaging and Morphometry at 7T Using MP2RAGE. *PLoS One*. 2014;9(6):e99676.

7. Fischl B. FreeSurfer. *NeuroImage*. 2012;62(2):774-81.

8. Alexander-Bloch A, Giedd JN, Bullmore E. Imaging structural co-variance between human brain regions. *Nat Rev Neurosci*. 2013;14(5):322-36.
